# Overwhelmed and Unnoticed—Burnout in Biomedical Scientists: A Narrative Review

**DOI:** 10.3390/ijerph23080963

**Published:** 2026-07-25

**Authors:** Molly J. Taylor, Tait D. Shanafelt, Lisa G. Williams

**Affiliations:** 1 Office for Organizational Well-Being, University of Kentucky College of Medicine, Lexington, KY 40536, USA; lisa.williams@uky.edu; 2 Stanford Medicine, Stanford University, Stanford, CA 94305, USA; tshana@stanford.edu

**Keywords:** occupational stress, academic medicine, faculty, researchers

## Abstract

**Highlights:**

**Public health relevance—How does this work relate to a public health issue?**

- Burnout is a recognized crisis amongst the healthcare workforce, driving attrition, stressing healthcare systems, and directly impacting patient care.
- Biomedical scientists/academic medicine research faculty and staff are critical to this workforce and the advancement of human health, but their experiences of occupational burnout are understudied with mitigation and intervention efforts lacking.

**Public health significance—Why is this work of significance to public health?**

- Biomedical scientists experience comparable burnout rates to physicians and clinical faculty with fewer resources; the subsequent ramifications and impact on healthcare infrastructure are poorly understood.
- There is a disproportionate focus on frontline healthcare workers in the literature; the root drivers and public health implications of biomedical scientist burnout warrant increased exploration and attention.

**Public health implications—What are the key implications or messages for practitioners, policy makers and/or researchers in public health?**

- To support biomedical scientist well-being and their contributions to public health, academic medical institutions should design interventions and systems to facilitate collegial relationships, protect time for teaching, revitalize passion and meaning in the work, support autonomy for those in PI roles, and explore feasibility of recommendations from stakeholders with improved collaboration and communication.
- Research and intervention efforts remain asymmetric between clinical and research faculties. Addressing this gap should start with engaging biomedical scientist faculty and research personnel in conversations to identify local challenges and ideate solutions, establish the associated costs of researcher burnout to academic medical enterprises to enhance buy-in for mitigation efforts, and design and evaluate the effectiveness of organizational interventions to improve the research work environment.

**Abstract:**

Biomedical scientists/researchers are critical to advancing human health, but their experiences of occupational stress, burnout, and subsequent consequences are surmised but understudied. The current narrative review aimed to search and summarize existing literature and scientific discourse specific to biomedical scientist burnout to better understand the prevalence and unique drivers of stress in this population and highlight critical areas that require further research and intervention efforts. The authors searched six primary databases from August 2024 to December 2025 using defined search terms to identify peer-reviewed articles and scientific discourse related to occupational burnout of academic medicine biomedical faculty researchers, postdoctoral researchers, or lab personnel. Prevalence and primary drivers of burnout, stress mediators, and mitigation/intervention recommendations were extracted from the included works. A total of 16 empirical articles and 4 non-empirical works were included. Over 30% of biomedical scientists exhibit high occupational burnout, nearly 45% report high distress, and just over 34% experience high emotional exhaustion. Seven primary drivers of biomedical scientist distress were identified including intense workloads/expectations, funding pressures, and lack of perceived organizational support/recognition. Biomedical scientist burnout prevalence appears comparable to clinical faculty but is driven, in part, by distinct stressors. The attributable organization and societal cost of biomedical scientist burnout remain unquantified. The current review establishes a significant need for further research and tailored efforts to address the distinct needs of biomedical scientists across academic medical centers.

## 1. Background

Biomedical scientists and researchers play a critical role in medical systems and the advancement of human health, yet their experiences of occupational burnout and the subsequent ramifications are understudied and overlooked. Contributions of biomedical scientists have transformed fatal diseases into preventable and manageable disorders, made life-saving surgical interventions commonplace, and continuously inform our understanding and ability to prevent, treat, and cure human disease [1]. Burnout, an occupational syndrome characterized by emotional exhaustion, depersonalization, and low sense of personal accomplishment, is well studied across the majority of healthcare spaces [2]. Academic medical centers, which serve as workplaces for many researchers, are instituting broad reforms to mitigate the current burnout crisis affecting healthcare personnel and clinical faculties, but have largely disregarded the stressors and needs of researchers [3,4]. To ensure these efforts are distributed more equitably across these centers, we must first explore and understand the specific nature of burnout experienced by biomedical scientists and researchers.

Despite their critical role, researchers are rarely in the limelight, and their occupational experience is largely unexplored [5]. Higher-education faculty well-being, retention, and intent to leave have been evaluated since the 1990s, but little is reported concerning the unique dynamics and challenges experienced by the biomedical scientist faculty of academic medical institutions [6,7]. Few studies have evaluated the prevalence, impact of burnout, or need for mitigation efforts in biomedical scientists [2,5,8]. In academic medical centers, patient-facing clinical faculties have been the focus of many intervention efforts with minimal attention to addressing the needs of the biomedical scientist faculty, professional research staff, and trainees (e.g., postdoctoral fellows) [4,6,9,10].

Concerns for the well-being of multidisciplinary scientists have been voiced in a number of editorials, commentaries, and reviews in recent years [11,12,13,14]. In 2025, growing threats to US scientific infrastructure and the academic health research establishment have heightened these concerns and emphasize the urgent need to understand the experiences of biomedical scientists and their research teams [15,16,17]. Nonetheless, repeated calls for more research on biomedical scientists’ burnout remain primarily unanswered [8,18]. Overall, the number of physician and nurse burnout-related articles published in medical and allied health journals has more than doubled since 2018, vastly outnumbering the limited articles in biomedical scientist populations by a ratio of more than 30:1 [2,3,19].

Given the known complexities, competitive nature, and distinct demands of biomedical research, it is imperative that we gain insights regarding the current state, drivers, and consequences of burnout in biomedical scientists. By focusing on burnout, a widely studied phenomenon, the authors seek to expose a foundational gap in the research and lay the groundwork for an increased understanding of the factors that impact stress and workplace well-being in biomedical scientists. A 2024 review highlighted factors impacting academic faculty physicians and emphasized the gap in knowledge concerning key burnout drivers deriving from research and education demands among biomedical scientist faculty members [6]. Paradis et al. explored burnout in academic physician K grant awardees, but little could be extrapolated concerning the unique research-related contributors to overall burnout experiences [20]. The financial ramifications and consequences of physician burnout on medical systems are well documented and understood, exceeding USD 4.6 billion annually, but little is known about the toll of researcher burnout on organizational health of academic medical centers [3,10,21]. Determining the cost of biomedical scientist burnout may enhance institutional recognition and improve intervention efforts to address this issue. With knowledge regarding occupational distress in biomedical scientists lagging, these mitigation efforts across academic medical campuses and research centers have focused on clinical faculty members, leaving a large percentage of the workforce vital to the greater healthcare system overlooked and potential VOI (value on investment) for biomedical scientist-focused efforts unknown.

## 2. Aims

The current study provides a narrative review of burnout and occupational distress among biomedical scientists and research personnel including faculty, postdoctoral researchers, and professional research staff. For the purpose of this study, the term ‘biomedical scientists’ (BSs) is used to refer to faculty members without clinical responsibilities and staff researchers working within academic medical institutions or colleges of medicine. The aims of this review are to (1) broadly search and summarize existing literature and scientific discourse specific to biomedical scientist burnout to provide a foundational understanding of the prevalence and unique drivers of stress in this population and (2) establish a framework for a more rigorous review by gathering context, refining language, and identifying critical areas that require further research and intervention efforts.

## 3. Methods

The current review was initially pursued as a systematic review in partnership with a medical librarian; due to the limited number of empirical studies specifically evaluating biomedical scientist/faculty burnout and lack of standardized terminology to identify and define the population of interest, the systematic approach was unfeasible for the current effort and procured an unmanageable number of irrelevant articles. Given this fact, a narrative review was pursued to provide a baseline description of the literature, including the incorporation of expert opinions and commentaries to help further define the current state [22,23]. The current review begins to characterize biomedical scientist burnout, establishes a need for language/title refinement in this population, and lays the groundwork for future, more rigorous systematic reviews of the topic as additional primary data become available.

PubMed, Medline, Web of Science, Google Scholar, Embase, and PsycINFO were searched for peer-reviewed, English-language articles with no publication date restrictions. These indexed databases include discipline-specific and gold-standard platforms for a feasible, wide-reaching footprint. The search was conducted from August 2024 through December 2025. Variations in the search terms ‘burnout’, ‘well-being’, ‘faculty’, ‘research staff’, ‘basic science*’, and ‘biomedical science*’ were used. Additional articles were included from the reference lists of retrieved articles. Due to the sparsity of peer-reviewed publications and interest in non-peer-reviewed scientific discourse, expert opinions articulated in commentaries, editorials, and scientific magazine columns were included to amplify and diversify the discussion of the phenomenon. These streamlined inclusion criteria allowed the authors flexibility and feasibility for building an introductory perspective of the current topic. Every source was reviewed and included if it specifically examined or discussed factors related to occupational burnout or well-being of non-patient facing faculty researchers, postdoctoral researchers, or professional research staff/lab personnel in biomedical sciences. The motivators, stressors, and contributors to burnout and professional fulfillment amongst faculty physicians are well cited in the literature [10]; the current review aims to better define these factors amongst researchers operating in the non-clinical sectors of academic medical systems. Findings related to clinical faculty or academic physicians were not included in the current review unless they provided comparative analysis between clinical and biomedical scientist faculty groups or served to illustrate a relevant theme or discrepancy with biomedical scientists. The primary author (M.J.T.) independently extracted the relevant data, including (1) quantitative findings of empirical studies, (2) drivers of burnout organized by theme, (3) stress mediators or motivating factors, and (4) mitigation strategies. Two authors performed the critical review and editing (T.D.S. and L.G.W.).

## 4. Results

A summary of the primary empirical studies (*n* = 16) and quantitative findings on occupational distress and burnout in biomedical scientists is provided in Table 1 with perspectives and editorials (*n* = 4) summarized in Table 2. While limited, these works begin to provide insights into the experience of scientists. The authors condensed the extracted data into primary themes encompassing the most heavily discussed drivers: high-intensity workloads and expectations, administrative burden, funding pressures and hyper-competition, bureaucratic culture and/or corporate mindset, lack of perceived organizational support or recognition, relational conflicts (multi-level: peers within institution, organization leadership, individuals outside institution), and diminishing academic career opportunity.

Amidst the challenges and factors that contribute to distress, researchers also identified several mediating factors that motivate continued efforts in academic medicine including: collegial relationships, teaching, passion and meaning in the work, and autonomy for those in PI roles [25,30]. The complete summary of all extracted data concerning the drivers of burnout in biomedical scientist faculty members and lab personnel are summarized in Table 3.

## 5. Discussion

The 2023 AAMC (Association of American Medical Colleges) StandPoint™ Survey gathered engagement data from 15,354 clinical faculty and 2378 biomedical faculty scientists, demonstrating 31.2% of female biomedical scientists and 22% of male biomedical scientists reported at least some degree of burnout [27]. The Standpoint Survey demonstrates sustained higher burnout rates in clinical than biomedical scientists, but the current review demonstrates the need for increased attention to biomedical scientists [27]; nearly 45% of biomedical scientists in a 2023 study had high distress scores on the 9-item Well-Being Index (WBI), another demonstrated that nearly every participating biomedical scientist faculty member had at least a moderate level of burnout [24,25]. Iranian researchers demonstrated that exhaustion was the only Maslach Burnout Inventory (MBI) dimension where clinical faculty members scored significantly higher than biomedical scientists, suggesting similar concerning levels of cynicism and a low sense of personal accomplishment in both faculty groups [31]. Another study from Iran utilizing the MBI demonstrated that 54.1% of biomedical scientists exhibited moderate-to-high emotional exhaustion and 30.3% exhibited high burnout [28]. Additionally, the impact that decisions and orders made at the federal level may have on the prevalence and severity of high stress and burnout amongst scientists/researchers is unknown. A number of accomplished academic scientists have written opinion and perspective pieces emphasizing the intensifying challenges scientists are experiencing, the lack of research on the scientist experience, and the need for further study [8,18,39,40].

Existing explorations of burnout in academic researchers across non-medical disciplines highlight hypercompetition for funding, inadequate protected time for research, bullying or harassment, and organizational politics and/or bureaucracy as some of the many contributors to toxic work environments and job dissatisfaction [11,12,13]. The current review’s findings mirror and expand upon these factors with the discussion focused on the most heavily cited drivers of burnout in research populations.

Workload Volume and Administrative Burden: The complex and competing demands of biomedical scientists’ research result in long hours, weekend work, and overwhelming expectations. A sample of nearly 400 biomedical scientists demonstrated an average of 54.7 hrs. worked per week and fewer vacation days taken compared to clinical faculty members [36]. These findings reveal comparable hours to physicians: while hours vary widely amongst clinical specialties and 40.7% of physicians report working ≥55 hrs./wk., the national average rests at 50.8 hrs./wk. [41]. Nature’s 2021 Salary and Job Survey demonstrates the rising levels of work-related stress amongst academic scientists of all disciplines with >30% working >50 hrs./wk. [42]. A mixed sample of biomedical scientists and postdocs reports even higher prevalence of high-intensity work weeks, with 37% reporting >50 hr. work weeks and 12% reporting >60 [35]. Additional findings suggest an inherent assumption of high-intensity workload in academia; researchers report a universal expectation to work overtime hours and prioritize work over personal life in order to advance in their careers or achieve promotions amidst infinitely increasing standards [14,18,24,25,35]. Additionally, researchers note the challenge of taking time off with pressure to keep their labs running 24/7 to maintain continuity in their research output, be the first to publish, and stay competitive in the funding race [30]. Administrative tasks and regulatory responsibilities (e.g., financial management of grants, IRB and other regulatory aspects, human resources tasks/management of research team) are also reported as a large contribution to being overwhelmed; biomedical scientists commonly describe frustration with the volume of these distracting tasks and unyielding deadlines with inadequate support resources [8,18,39,40]. The AAMC StandPoint™ Survey corroborates these findings with nearly 1/3 of biomedical scientists reporting too much time spent on administrative duties and too little time on research [27].

Funding and Hypercompetition: Nestled within the challenge of high workloads exist the pressures of earning and maintaining funding. Acknowledged as one of the leading contributors to hypercompetitiveness and stress in research positions, the rising costs of research and falling rates of funded applications result in relentless grant application writing [8,33,39,40]. Contrary to the employment model for clinical faculty members (whose funding largely comes from patient care-related revenue), many biomedical scientists have a constant fear of failing to maintain sufficient funding to keep their positions and sustain their careers [8,18,31]. The responsibility to cover a portion of their salary—often greater than 50%—via grant funding is a constant source of stress and worry [8,30,39,40]. In addition, grant funding often supports the salaries of research staff, postdocs, and doctoral students who operate labs; faculty members feel responsible for the careers and livelihood of these individuals, augmenting funding-related stress [29,30].

Culture: Several works demonstrate researcher perceptions of a shift to a corporate, profit-driven mindset as well as bureaucratic institutional cultures [18,24,30]. This culture shift has resulted in productivity targets and the prioritization of the clinical enterprise (given the associated revenue) and pressure to develop research with commercial applications. Biomedical scientists also report distrust and conflict with institutional leadership due to a lack of communication and exclusion from decision-making [8,18,24,30]. Additionally, the competitive external environment is replicated internally in many institutions with reports of negative ramifications on the culture/community of departments [8,39,40]. These themes were expressed in a 2012 study of academic medicine culture and intent to leave, highlighting ethical conflicts and the nonrelational culture of the workplace as contributors to dissatisfaction [43].

Organizational Support and Recognition: Consistent with the findings across diverse work settings, including medicine, the literature on scientists indicates that feeling supported, acknowledged, and recognized by one’s organization significantly impacts well-being, work distress, and intent to leave [24,43,44]. Amongst biomedical scientist and lab personnel, perceptions of low organizational support are associated with high distress [24]. Additionally, lack of appreciation and acknowledgement at the institution level as well as within the social structure of academic medicine (unequal social status allotted to clinical faculty members relative to biomedical scientists) were demonstrated as leading drivers of exhaustion and burnout [25]. Biomedical scientist faculty members described perceived social discrimination with common reports of feeling ignored, unvalued, and treated as inferior to clinical faculty members. Scientists often cited external publications, grants, and conference appearances as their only experiences of recognition [8,25,29,30]. Lack of resources including support personnel, number of faculty members, childcare services, outdated equipment and facilities, and lack of infrastructure or efficient procedures to modernize also diminished perceptions of support and recognition [25].

Relational Conflict: Nearly 52% of biomedical scientists in a 2019 study reported client-related burnout due to interactions with administrators, colleagues, funding agencies, students, or other work relations [33]. This displays that while biomedical scientists may not be patient-facing, their roles involve complex and demanding human interactions that contribute to their burnout experiences. These client-related stressors were significantly higher in biomedical scientists than all other healthcare professionals (25.6% in nurses, 18.6% in physicians, 15.1% in residents) [33]. Conflict with both colleagues and supervisors has been demonstrated to have a significant association with high distress scores, with 5x greater odds of distress in biomedical researchers/personnel experiencing conflict with their supervisor [24]. Even more concerning are the abusive interpersonal interactions reported in hierarchical science environments. In 2015–2019, surveys suggested that 25–35% of multidisciplinary academicians in research had been bullied or harassed in the workplace in the past year, with 40–50% witnessing bullying second-hand [11,45]. A 2018 study report on sexual harassment in academic sciences from the National Academies (NASEM) defines the conditions that increase the risk of sexual harassment perpetrated against women, including organizational tolerance, male-dominated environments, hierarchical relationships between faculty and trainees, and isolating environments (e.g., labs), all of which are common in biomedical scientist environments in academic medicine centers [46]. A 2024 study demonstrated that female academic medicine faculty members (mixed sample of biomedical scientists and clinical faculty members) experienced gender-based discrimination and harassment at 11 and 5.7 times the rate of men, respectively, with 28.1% reporting discrimination or harassment in the previous year [34]. The true prevalence of workplace bullying specifically between biomedical scientists in academic medical institutions is unknown and difficult to quantify, yet the abusive behavior of senior researchers towards trainees is a problem acknowledged as deeply entrenched in the academic world [40]. Researchers studying the influence of effective mentorship on academic medical faculty vitality determined that a one-point improvement in the mentoring quality score was associated with a 30% reduction in serious intent to leave academic medicine [5]. Some researchers seeking a more positive dynamic or collegial working environment have migrated away from academic medicine, reporting more genuine teamwork among colleagues in industry positions [40].

Academic Career Opportunity: The previously outlined drivers of burnout among biomedical scientists are amplified by a worry about the sustainability of academic biomedical research as a career. Most high productivity biomedical laboratories function on the work of graduate students and postdoctoral fellows, with many principal investigators training more scientists than needed to step into their faculty shoes [35,39]. This creates an environment of scarcity: a large supply of trained scientists with few open academic positions and funding opportunities to pursue [43]. Strikingly, 88% of biomedical scientist faculty members report an increased prevalence of trainee discouragement about future career opportunities [8]. Many graduate students and postdoctoral fellows view academic medicine as a ‘pyramid scheme’ for basic scientists with relentless competition for a dearth of jobs [35]. Countless highly trained postdoctoral fellows remain in underpaid trainee positions for 3+ years with perceptions of stagnation, lost hope, and intent to leave academia [25,35,40]. The perception of limitations on career longevity or advancement extends beyond students and postdocs: PhD scientists reported low self-efficacy in career advancement at double the rate of clinical faculty researchers (49% vs. 24%, respectively) [5].

Mediators: Exploring stress mediators and motivational factors is crucial to understanding well-being and mitigating burnout. Biomedical scientists have cited teaching, appreciation from students, and supporting student success as positive aspects of their work and mediators of work-related distress [25,30]. Additionally, colleague support and pride in one’s research and achievements related to human health are strong contributors to job satisfaction and sense of meaning [25,30]. For principal investigators, autonomy, independence of thought, decision-making, and flexibility contribute to better working conditions and overall well-being [30]. Drivers and mediators of work stress both contribute to work environments, job satisfaction, and well-being, and must be part of holistic explorations and intervention efforts moving forward.

**Table 3:** Drivers of occupational burnout in biomedical scientist faculty and research personnel.

Primary Theme	Subthemes/Examples
Workload and Expectations	Long hours, high workload [18,24,32]. Of 79 biomedical scientists, 66.2% reported ‘spending too many hours at work’ [37]. Of 30 biomedical scientist PIs, 17% reported working >70 hrs. in the preceding week; 25% reported working a 7-day week [35]. Average hours worked (*n* = 394) reported at 54.7 h per wk. [36]. Teaching is a high-level job expectation with no compensation [30]. Competing workloads: teaching and complex demands of research [35]. Increasing standards for academic excellence; unattainable productivity goals (more grant funding, higher quality publications, and higher teaching and collaboration expectations) [18]. Hypercompetition involved in attaining and maintaining a faculty position demands sacrifice of work–life integration [35]
Administrative Burden	Administrative and regulatory tasks: time spent; heavy burden [8,18,39,40]. Frustration over administrative duties [8]. ‘We are caught up in so many minute nonacademic things in the department [plus personal life stressors] that we never get any protected time to do and work on our research projects.’ [32].
Funding	Funding pressures; hypercompetition [8,33,39,40]. Hypercompetition suppresses creativity, cooperation, risk-taking, and original thinking (system favors guaranteed results over path-breaking ideas) [39]. Funding challenges: fear of not maintaining sufficient funding to keep position, sustain career, and support the salary and livelihood of the research team [8,29,30]. Reliance on grant funding: often >50% of salary [30,39] (achieving tenure is not protective for biomedical scientists if large portion of salary still relies on soft funding; feel like ‘independent contractors’) [30]. Insufficient compensation also listed as top stressor among biomedical scientists [37]. Of 79 biomedical scientists, 38% of those who identified research as the most exasperating aspect of their job listed obtaining research grants as the primary contributor [37]. Less funding for biomedical sciences: push for more translational research [39,40]. Rising cost of research paired with falling % of applications funded: supply/demand mismatch (faulty assumption that biomedical research system expansion is limitless; expansion has stalled and even declined over the last decade) [39].
Bureaucracy and Leadership Conflict	Faculty distrust in enterprise due to perceived adversarial relationship with senior leadership [8]. Power shift in autonomy and decision-making from faculty to administration [8]. Lack of communication; faculty members feel disenfranchised from decision-making [8]. Faculty–administration value alignment issues and conflict [18]. Conflict with local supervisor/leadership [8,24] (conflict with supervisor associated with highest increase in odds of work distress) [24]. Lack of participatory leadership; lack of action/response to faculty feedback [30]. Of 79 biomedical scientists, 70.6% reported excessive regulation by the government or university [37].
Institutional Inefficiencies	Dysfunctional organization structure (e.g., work processes) and mismanagement (delays and reductions in expected payment): leading cause of fatigue in biomedical scientists in a comparative study between biomedical scientists and clinical faculty members [31]. Issues with faculty retention and turnover: loss of valued colleague [30]. Grant/salary mismanagement (i.e., administrative decisions or policies that clash with pre-established fund appropriation); lack of understanding of funding mechanisms and associated policies [30]. Lack of transparency, lack of clarity concerning institutional objectives, and sub-optimal communication [30].
Culture	Shift to corporate mindset: focus on productivity targets; commercialization of science [8]. Pressure to pursue research with commercialization potential [18]. Perceived prioritization of clinical enterprise/mission [30]. Hypercompetitive internal environment: resources, promotions, and pressure from institution/chair intensifies comparison and implicit competition with colleagues [8,18,39,40].
Organizational Support and Resources	Lack of perceived support from organization/executive leadership [24,25]. Lack of institutional appreciation and acknowledgement of the contributions of scientists [25]. Sub-optimal infrastructural support for scientific work: outdated equipment, lack of physical space [29,30], and lack of advancement opportunities [25,29]. Lack of support resources: low faculty numbers; lack of supportive facilities (childcare) [25]. Inability or lack of organizational support to take time off [24].
Faculty Disparities and Recognition	Focus on clinical faculty; health and well-being of biomedical scientists ignored [8]. ‘Social discrimination’: unequal status compared to clinical science faculty; feel like second-class citizens [25,30]; ‘…[my family has] a bias against teaching in basic sciences. The main frustration is that no one considers you a doctor. You are doing a lot, but still no one considers you anything.’ [32]. Biomedical scientists do not feel valued or recognized (only recognition is external: publications, grants, awards, and conferences) [29]. Gender disparities: women expected to take on greater teaching and committee loads, threatening advancement opportunities [30], fewer grant awards and less funding to women who are awarded, credited less for research contribution s [40], and familial expectations heavier on women [32].
Relational/Interpersonal Conflicts	51.5% of basic scientists reported ‘client-related’ burnout due to tension with colleagues, funding agencies, students, administrators, and others [33]. Mistreatment: senior researcher behavior towards trainees [40]. Female faculty members who experience gender-based discrimination or harassment demonstrate increased odds of burnout (OR = 1.65) [34]. Inequity between senior and junior faculty members: higher work expectations on junior faculty [25]. Of 79 biomedical scientists, 40.5% of those who identified research as the most exasperating aspect of their job listed conflict between researchers as the primary contributor [37].
Diminishing Career Opportunity in Academia	Environment of scarcity: competition for limited number of jobs [35]. Dearth of career opportunities: 88% of biomedical scientists reported increases in graduate student/postdoc discouragement about future career opportunities [8]. Unequitable pay/compensation for biomedical science postdoctoral fellows/researchers [40]. Stagnation: lack of hope of progressing in career [25]; concern over uncertain future for young investigators [18]. Lack of opportunity for advancement: 82% of biomedical scientist faculty members who had left their academic jobs cited lack of professional/advancement opportunities as primary contributor [29]. 49% of PhD scientists reported low self-efficacy in career advancement vs. 24% of physician investigators [5]. Nearly 1/3 of surveyed PhDs, postdocs, and PIs reported intent to leave academic research primarily due to a lack of job opportunities [35]. Industry training unavailable for doctoral candidates; industry experience undervalued for candidates seeking academic/faculty appointments [40].
Mediators/Motivating Factors	Teaching: student interaction, gratitude, acknowledgement, and success [25,30,32]. Co-worker/colleague support and collegial relationships [5,25,30]. High-quality mentoring, strong work relationships, and feelings of inclusion and trust all independently linked to reduced consideration of leaving academic medicine [5]. Positive perceptions of direct supervisor: higher MCLI (Mayo Clinic Leadership Index) ratings associated with reduction in biomedical scientist faculty member burnout [34]. Satisfaction from the work/research itself and achievements associated with that research [30]. Sense of meaning and pride in one’s work; making discoveries that help/benefit people and human health [30]. Autonomy: independence of thought, flexibility/decision-making (PI’s with own lab) [30]. Mixed sample of clinical and biomedical scientist faculty members: those who spent more time with their families were less likely to report low well-being (OR = 0.44) and burnout (OR = 0.26) [28].

With intervention efforts to address burnout in biomedical scientists lacking, resignation and ‘quiet quitting’ rates continue to rise [12,47,48]. This phenomenon is manifest via reducing participation at conferences, limiting committee membership, and reducing efforts related to peer-review, mentoring, teaching, outreach, and inclusivity [48]. Researchers and educators have shared numerous mitigation tactics and requests for action regarding the funding crisis, bureaucratic shifts, lack of recognition and organizational resources, and diminishing academic career opportunity. The current review summarizes these recommendations while simultaneously recognizing the complexities and barriers to implementation of many of the offered strategies and tactics. Counterarguments or explorations of these barriers are not included. A complete summary of identified solution recommendations from the included works are outlined in Table 4.

Stakeholders and researchers familiar with the stressors of basic science and biomedical research suggest that institutions focus on harm-reduction measures: increase bridge funding to support struggling faculty members, reduce the salary that faculty members are responsible for covering through grants, and improve administrative support for the grant submission process [8,18,30]. In the last decade, the National Research Council (NRC) suggested that the federal government should adopt stable policies, practices, and funding for university-performed research as well as create a Strategic Investment Program that specifically funds university research initiatives [8]. To date, this level of strategic attention to funding has not been prioritized.

Bullying, harassment, and unacceptable behavior occurrences in biomedical science settings are often shrouded in secrecy, shame, and disagreement in expectations across disciplines with no universally accepted guidelines for how to manage and respond to reported cases [8,13,25,33,39,40]. Bullying and harassment are similarly defined and often grouped, respectively defined as persistent or threatening physical behaviors or verbal abuse related to some sort of hierarchical disadvantage and threatening, harmful, or humiliating conduct based on race, color, national origin, sex, or disability [49]. Nature’s 2021 Global Salary and Job Satisfaction survey demonstrated that 27% of 3200 respondents reported observing or experiencing discrimination, bullying, or harassment in their present position with 25% of postdocs personally experiencing discrimination and harassment [13]. While the exact prevalence of confirmed harassment and discrimination in academia is unknown, the NASEM report estimated that more than half of female faculty members and staff have encountered or experienced sexual harassment [13]. Researchers call for heightened attention to these experiences, including increased transparency, institutional actions, mandatory disclosure of behavioral misconduct, and national efforts such as worldwide professional certifications that can be revoked if ethical violations occur [13]. Some calls have been answered; in 2022, the National Institutes of Health (NIH) outlined new policies and requirements of NIH recipient institutions to foster an environment free from harassment, including, but not limited to, sanctioned codes of conduct, accessible and protected processes for reporting harassment, and requirements to notify the NIH when senior personnel are removed or disciplined due to harassment allegations [50]. Future research should explore the prevalence, impact, and management of bullying, harassment, and discriminatory behaviors in the biomedical scientist realm.

The common perceptions amongst biomedical scientists of not feeling valued, appreciated, supported, or acknowledged speak to the need for organizational leaders to demonstrate a recognition of scientists within academic medical centers [24,25,29,30]. To address perceived bureaucratic processes and conflict between faculty and leadership, researchers from the MD Anderson Cancer Center in Houston suggest starting with a framework of programmatic efforts to engage scientists [18]. This starts with listening and utilizing a faculty senate or similar structure to collect input from biomedical scientists about sources of faculty stress and their ideas about pragmatic initial approaches to mitigate them [18]. Multiple sources highlight the opportunity for more collaborations, including joint troubleshooting, communication between faculty and executive members, and alignment regarding institutional values [8,18,29].

To address the reality that there are many more trained PhDs than faculty scientist positions available, researchers suggest offering continued transparency and assistance in transitioning PhDs and postdocs to career opportunities outside of academia [8]. The US National Science Foundation (NSF) survey of earned doctorates demonstrates steady progress: in 2022, 48.1% of individuals who earned doctorates reported employment in industry positions [51]. Researchers also suggested that an increase in permanent laboratory staff positions may help offset the overpowering ratio of temporary trainees to staff [24,39]. Researchers note some improving standards: the NIH increased the minimum starting postdoctoral salary by 8% in 2024 requiring USD 61,008 per year, setting a precedent that institutions are encouraged to meet [52]. Additional approaches to foster career opportunities and advancement include improving faculty training/mentoring programs and implementing adjusted service structures and research collaboration acknowledgements within biomedical scientist faculty appraisals and criteria for promotion [18,25].

The strengths and weaknesses of leaders and supervisors directly impact work communities, with leader qualities proving to be highly impactful on the individual well-being, burnout, and satisfaction of those they supervise [53]. The findings of this review amplify this statement, demonstrating greatly increased odds of distress and dissatisfaction in biomedical scientist researchers experiencing conflict with a supervisor, and conversely, a reduction in burnout amongst those with more positive perspectives of their supervisor [24,34]. A Nature correspondence highlighted the often underemphasized contributions to burnout from local leadership/management, noting the need for a shift of focus to effective team management vs. research production alone [45]. Practices to enhance leader self-awareness, including professional development and leadership training or coaching, can have positive ripple effects throughout departments and systems [54,55,56].

While organization-based and structural strategies must be prioritized, researchers and authors indicate that individual-focused tactics to support scientist well-being should also be explored and developed. Social connections and community at work have been demonstrated as vital to well-being and longevity, both personally and professionally [57,58]. The hypercompetitive environment fueled by limited funding opportunities and a ‘publish or perish’ culture can complicate scientists’ relationships at work, yet researchers desire improved collaboration, mentorship, and teamwork [8,40].

Finally, biomedical scientists are a resilient population, with individual resilience accounting for less than 10% of the variance in levels of distress in biomedical researchers [24]; resilience alone cannot prevent or protect against systemically-derived contributors to stress and burnout in academic medicine [24,59]. Putting energy into personal health is essential and may look different between faculty groups: a 2006 study demonstrated that biomedical scientists discussed the stressful nature of their work less frequently with loved ones than their clinical counterparts [36]. While data from that study is dated, exploring these subtleties of well-being and mediating strategies is crucial in addressing burnout moving forward.

A narrative review is less reliable than a systematic or scoping review with a higher risk of bias, restricted repeatability, and a less systematic search strategy. However, due to the limited number of empirical studies evaluating the occupational experience of scientists and the lack of standardized terminology to identify and define the population of interest, a systematic search using exhaustive terminology procured an excessive number of irrelevant articles that did not fit the inclusion criteria. Given the limited number of empirical studies, the narrative review format also allowed for the incorporation of expert opinions and commentaries to help further introduce the current state and provide a foundation for future efforts. While the search strategies used for this review were wide-reaching and repeated over many months, relevant articles or opinion pieces may have been overlooked and not included. Multiple included works were published over 10 years ago and may not fully reflect the current state of scientists’ occupational experience. Although the narrative review provides less rigor and generalizability relative to a systematic review, it nonetheless provides a practical synthesis of the current state of the literature to guide future research [22]. Several of the included studies were conducted or published in countries other than the United States. While some aspects of this review may be context specific and not be generalizable to all research context around the world, we suspect the findings relative to workloads, funding and publication pressures, recognition, and resources/perceived organizational support have at least some universal relevance [8,24,25,31,32,33,39,40]. Finally, although the search included manuscripts published in December 2025, it remains a dynamic time in the environment (e.g., federal overhaul of the funding environment and politicization of biomedical science) in which researchers function with new challenges emerging.

## 6. Conclusions

Further research is needed to scrutinize the identified drivers of stress and occupational burnout in biomedical scientists, define the attributable cost of biomedical scientist burnout to academic medical centers, explore feasible multi-level mitigation strategies, and provide more empirical data on the professional experience and needs of this understudied population. Academic leaders interested in addressing this gap should engage biomedical scientist faculty and research personnel in conversations to identify local problems and ideate solutions from the ground up. Research into the associated costs of researcher burnout to academic medical enterprises is necessary to enhance the investment to improve occupational well-being among biomedical scientists across academic departments. Evidence-informed interventions specifically designed to mitigate burnout and/or foster occupational well-being among biomedical scientists are limited. The design, implementation, and evaluation of organizational interventions to improve the work environment for biomedical scientists should be a high priority for academic medical centers. Additionally, the authors recommend that further publications related to this work utilize or make reference to similar language and titles defined in the current review to better identify the population and facilitate research collaboration and review processes moving forward. Specifically, the authors recommend the term ‘biomedical scientists’ be used as an inclusive term that encompasses research scientists in both basic science (e.g., biochemistry, genetics, cell biology) and biomedical sciences (e.g., biostatistics, epidemiology). The additional language to specify whether the biomedical scientists studied work in an academic context (i.e., academic faculty) or non-academic context (e.g., industry settings) should be specified.

For years, vanguard organizations including the Office of the Surgeon General, National Academies, Mayo Clinic, and Stanford Medicine have emphasized that healthcare worker burnout cannot be mitigated solely through individual or local efforts and must include a system-level approach with institutional buy-in [2,19,60]. These and other organizations have pioneered the implementation of workplace well-being frameworks with proven efficacy in healthcare spaces. To a large extent, these recommendations have yet to be applied specifically to address the occupational well-being of biomedical scientists [2,19,60]. It is time to address this issue and expand the efforts to improve occupational well-being across the entire academic medical workforce. This review summarizes the limited empiric data on the occupational well-being of biomedical scientists and establishes a significant need for greater attention, research, and tailored intervention efforts to support scientists and those who benefit from their discoveries.

## Figures and Tables

**Table 1:** Summary of empirical studies.

Details and Design	Outcome Measure(s)	Description and Quantitative Results
Boitet et al., 2023 [24] USA Cross-Sectional Survey	2-item Connor Davison Resilience Scale (CD-RISC2) 9-item Well-Being Index (WBI) Single-item Moral Distress Likert scale measure 3-item adaptation of the Perceived Organizational Support (POS) measure	Survey study exploring organizational drivers of distress among biomedical researchers/scientists at an academic medical center (*n* = 245) 43.7% exhibited high distress scores (WBI ≥ 2) Variables with significant association to high distress: heavy workload/long hours (reported by 30.6% of scientists, *p* < 0.001), inability/lack of support for time off (14.7%, *p* < 0.001), conflict with colleagues (11.4%, *p* < 0.05), and conflict with supervisor (10.2%, *p* < 0.001) Low POS scores, CD-RISC2 scores, and high moral distress all significantly associated with WBI distress (*p* < 0.001) Odds ratios: increased odds of high distress score on WBI: heavy workload/long hours (OR = 3.25, *p* = 0.002), inability/lack of support for time off (OR = 2.80, *p* = 0.045), conflict with supervisor (OR = 5.03, *p* = 0.033), and resiliency (CD-RISC2) (OR = 0.69, *p* = 0.003) Regression: perceived organization support accounted for 27.89% of predicted variance in distress, followed by workload/long hours (18.39%), conflict with supervisor (13.08%), inability to take time off (12.86%), and resilience (9.97%)
Choudry et al., 2022 [25] Pakistan Mixed Methods	16-item Oldenburg Burnout Inventory (OLBI), Disengagement and Exhaustion subscales Interviews	Mixed-methods study exploring burnout and associated factors among BS faculty members from 3 medical institutes (public and private) (*n* = 113 surveyed, subset interviewed [those with moderate/high level burnout]) Avg OLBI scores: disengagement (2.24 ± 0.27 [moderate burnout]) and exhaustion (2.35 ± 0.24 [moderate burnout]) No difference in OLBI disengagement or exhaustion between scientists working in public and private sectors Primary qualitative themes (and subthemes) include: (1) lack of acknowledgement (appreciation for work, social discrimination), (2) lack of resources (lack of faculty, lack of infrastructure, lack of facilities), (3) motivational factors (student acknowledgement, co-worker support), and (4) Lack of Progress system
Dandar et al., 2019 [26] USA Cross-Sectional Survey	100+-item AAMC StandPoint™ Faculty Engagement Survey, 17 dimensions Single-Item Burnout Measure	Analysis in Brief of the 2019 AAMC StandPoint™ Faculty Engagement Survey. This survey is a professional service offered to academic medical institutions by the AAMC to assess factors related to faculty engagement and retention via assessment of burnout, intent to leave, job satisfaction, and more. The 2019 Analysis in Brief offers a summary of the primary faculty burnout findings from the 2016–2018 surveys administered to a convenience sample of 13 institutions (*n* = 7653: 6609 clinical, 1044 BS) Burnout by department type (% burning out/burned out): Basic Sciences: 26% Clinical (provide pt. care): 31% Clinical (no pt. care): 28% Among biomedical scientist faculty members, those in departments of epidemiology, biostatistics, and medical education reported the highest burnout (29%), followed by biochemistry (27%) and neuroscience (26%) Burnout correlated with overall workplace engagement across all faculties: satisfaction with department (0.418, *p* ≤ 0.001), satisfaction with school (0.399, *p* ≤ 0.001), and likelihood of staying at current institution (0.329, *p* ≤ 0.001)
Dandar et al., 2023 [27] USA Cross-Sectional Survey	100 + -item AAMC StandPoint™ Faculty Engagement Survey, 17 dimensions Single Item Burnout Measure	Aggregate of StandPoint™ Faculty Engagement Survey data in 2020–2022 for participating academic medical institutions exploring faculty engagement and retention (*n* = 17,733: 15,354 clinical, 2378 BS) Burnout: (% burning out/burned out): BS: 26.6% (women: 31.2%, men: 22%) Clinical: 32.5% (women: 38.4%, men: 26.5%) Engagement (% disengaged): BS: 7.9% Clinical: 8.7% Intent to leave (% highly unlikely/unlikely to leave in 1–2 yrs.): BS: 75.9% | Clinical: 74.1% Faculty members considering/planning to leave in 1–2 years reported significantly lower agreement on questions pertaining to their relationship with their supervisor, sense of belonging to their department, and satisfaction with opportunities for professional development (*p* ≤ 0.05)
Ghahramani et al., 2024 [28] Iran Cross-Sectional Survey	22-item Maslach Burnout Inventory—Human Services Survey (MBI-HSS), emotional exhaustion, depersonalization (DP), and personal accomplishment (PA) subscales: Persian translation 7-item Well-Being Index (WBI) Additional items including workplace violence and intent to leave	Survey study examining the prevalence and predictors of burnout and well-being in faculty members employed at Shiraz University of Medical Sciences. Note: study undertaken in 2021 during COVID-19 pandemic (*n* = 222: 113 clinical, 109 BS) MBI (mean scores): Overall burnout: Clinical: 23.8% Biomedical scientists: 30.3% EE: Clinical: (21.68) 50.4% moderate–high EE Biomedical scientists: (18.27) 54.1% moderate–high EE DP: Clinical: (2.65) 12.4% moderate–high DP Biomedical scientists: (2.04) 11.1% moderate–high DP PA: Clinical: (35.61) 69.9% moderate–high PA Biomedical scientists: (37.16) 71.6% moderate–high PA WBI (mean score): Clinical: (3.81) 58.4% low well-being Biomedical scientists: (3.10) 42.2% low well-being Faculty members who spent more time with their families were less likely to report low well-being (OR = 0.44) and burnout (OR = 0.26); faculty members expressing intent to leave were more likely to report low well-being (OR = 6.23) and burnout (OR = 2.97)
Girod et al., 2017 [29] USA Cross-Sectional Survey	Study specific Likert-type survey built from Faculty Attrition Survey (University of Wisconsin-Madison) and Faculty Career Survey (designed by author) and open-ended questions	Survey study exploring reasons for faculty departures from an academic medical center (*n* = 88: 47 Clinical, 38 Biomedical scientists). Topics explored included time use, perceived accuracy of stated and expected roles, work satisfaction, and perceptions of recognition and support 82% of Biomedical scientist faculty members cited lack of professional and advancement opportunities as the primary reason for leaving. Both Clinical and Biomedical scientists’ role attrition motivated mainly by external opportunities that provided more room to develop their research Biomedical scientists cited not feeling valued or recognized
Goranflo, 2017 [30] USA Qualitative Study (Dissertation)	Semi-structured interviews (using Hagedorn’s Conceptual Framework for Faculty Job Satisfaction)	Exploration of personal and professional factors that contribute to morale and job satisfaction in biomedical research faculty at academic health centers (*n* = 12) Factors related to satisfaction include recognition, autonomy, advancement opportunity, funding mechanisms/stability, gender, institution type, collegial relationships, quality of student relationships, perception of administration/institution values/priorities, business-model culture, funding, retention, and resources/space
Haghighinejad et al., 2021 [31] Iran Cross-Sectional Survey	16-item MBI General Survey (MBI-GS): EE, DP, PA subscales: Persian translation Likert-type questions related to job and income satisfaction, value, and causes of fatigue	Survey study comparing burnout between clinical and BS faculty members of a medical school (*n* = 225, 41 Biomedical scientists). Secondary aim to assess reliability and validity of Persian MBI Significantly higher exhaustion (EE) MBI scores in Clinical faculty (MBI-GS = 2.2) vs. Biomedical scientist faculty (MBI-GS = 1.44) (*p* = 0.017). No significant differences between groups in cynicism/depersonalization (DP) or professional efficacy/accomplishment (PA) dimensions Academic rank, work hours from home, total working hours per week, job satisfaction, income satisfaction, number of children, and Clinical vs. BS faculty had significant association to at least one dimension of MBI burnout Regression: job satisfaction (JS) and income satisfaction (IS) negatively correlated with exhaustion (JS *β* = −2.33 [*p* < 0.001], IS *β* = −0.88 [*p* = 0.002]), and cynicism dimensions (JS *β* = −1.83 [*p* < 0.001] IS *β* = −0.48 [*p* = 0.02]) and job satisfaction positively correlated with professional efficacy (*β* = 0.9 [*p* = 0.001] Primary causes of fatigue in both groups: workload/dysfunctional work structure (37%) and organization mismanagement (34%). Highest cause in Biomedical scientist faculty was organization mismanagement
Hashmi, 2021 [32] Pakistan Mixed Methods	9-item abbreviated Maslach Burnout Inventory (aMBI): 3-item versions of Emotional exhaustion (EE), depersonalization (DP), and personal accomplishment (PA) subscales Study specific qualitative survey	Mixed-methods study exploring burnout in medical teachers in a public medical university in Pakistan. 203 respondents completed the aMBI (134 Clinical, 68 Biomedical scientists); 10 exhibiting high burnout (EE score ≥ 9, DP scores ≥ 6) were selected for detailed semi-structured interviews exploring reasons for burnout (*n* = 10, 8 Clinical, 2 Biomedical scientists) Burnout: 38.9% of medical teachers reported high burnout on the EE subscale 31.5% reported high burnout on the DP subscale Qualitative themes related to experiences of burnout, both contributing factors and mitigating factors/strategies (4 categories): Work-related factors Sub-themes: high responsibility (personal responsibility, acuity of patients), work environment (high workload, work pressure, thoughts of leaving medical field, work schedule, lack of job description, workplace politics, work conditions, workload) Family or social factors Sub-themes: women’s challenges (family responsibility), family and social bias (particularly bias against teaching in BS), social life outside work (lack of) Feelings/Emotions Sub-themes: negative feelings (physical and mental fatigue, uncertainty/self-doubt, regret), positive feelings (gratitude/thankfulness from trainees/patients, equanimity, mastery) Personal qualities, habits, and preferences Sub-themes: positive (focus, meticulousness, personal improvement/accomplishment), negative (personal health, habits, appearance, privacy, personal distance)
Messias et al., 2019 [33] USA Cross-Sectional Survey	19-item Copenhagen Burnout Inventory, Personal, work-related, client/patient-related subdomains	Survey study exploring multidisciplinary burnout prevalence in a medical center (*n* = 1646, 108 BS). Total sample burnout prevalence: 52.7% *(95CI, 50–55%)* Mean CBI scores: Personal: Clinical: 48.9 *(95CI, 41.9–56.0) |* BS: 38.1 *(95CI, 28.1–47.4)* Work-related: Clinical: 43.8 *(95CI, 36.8–50.8) |* BS: 30.7 *(95CI, 21.6–39.7)* Client/Patient-related: Clinical: 18.6 *(95CI, 13.1–24.0)* | BS: 51.5 *(95CI, 41.6–61.4)* Biomedical scientists had highest mean CBI score in client-related burnout (45.4 ± 24.3), ‘client’ designated as follows: colleagues (33%), funding agencies (28%), students (18%), administrators (14%), and others (7%). Biomedical scientists increased odds of client-related burnout (AOR) = 10.0 (95CI 5.7–17.6)
Peccoralo et al., 2024 [34] USA Cross-Sectional Survey	2-item MBI: *EE*, *DP* 2-item Patient Health Questionnaire (PHQ) 2-item CD-RISC 1-item Work Life Integration (WLI) assessment 1-item Meaning in Work assessment 9-item Mayo Clinic Leader Index (MCLI): Direct supervisor Gender-based discrimination and harassment questions	Survey study exploring differences in burnout and gender-based discrimination and harassment among physician and scientist faculty members at a large urban academic medical institution. Additionally examined the relationship between burnout and gender discrimination to various occupational and personal factors (*n* = 1411, 139 BS) BS faculty demonstrated lower rates and decreased odds of burnout than clinical faculty members (OR = 0.53 [0.30, 0.93] 95CI) Women demonstrated higher rates of burnout than men (30.9% vs. 23.3%) Women experienced gender discrimination at 11 times the rate and harassment at 5.7 times the rate of men. Experiencing gender-based discrimination or harassment associated with increased odds of burnout in women (OR = 1.65 [1.04, 2.61] 95CI] For every 1-point increase in MCLI rating of supervisor, burnout decreased by 3.6% overall, 4% in women, and 3.2% in men
Pololi et al., 2023 [5] USA Cross-Sectional Survey	Study specific survey built from items from C-Change Faculty Survey (vitality: career advancement, research, work–life integration, quality of mentoring received) Single-item Burnout Measure Intent to Leave question Additional items exploring cross-cultural issues (identity, self-awareness, valuing diversity, anti-sexism, anti-racism)	Analysis of survey data gathered to identify factors related to academic medical faculty vitality and career accomplishments (survey deployed for baseline measurements for NIH funded randomized controlled trial testing a mentoring intervention in midcareer researchers at US academic medical centers). Included assistant or associate professors with demonstrated research success (current or recent first-time NIH R01 or R01 equivalent award, HRSA, ARHQ, or other federal agency major grant, K training grant, or recent major foundation or professional organization grant) (*n* = 146, 86 Biomedical scientists [PhD]) 75% of all faculty responded not at all, to a very small extent, or to a small extent when rating how well mentoring helped in assessing alignment between professional activities and personal values For every one-point improvement in mentoring quality score, there was a 30% (15–42%, 95CI) reduction in the probability of seriously considering leaving academic medicine 49% of PhD scientists reported low self-efficacy in career advancement vs. 24% of physician investigators For all faculty members, 40% (32–49%, 95CI) reported feeling burned out frequently or very frequently. Women were 4× as likely to report the max response option for burnout (very frequently) than men (20% vs. 5%) 24% (17–32%, 95CI) had seriously considered leaving academic medicine 12 months prior (no significant differences between PhDs, MDs, or PhD/MDs) Vitality strongly associated with intent to leave in all faculty members: for every one-point decrease in the six-point vitality scale, the probability of seriously considering leaving academic medicine more than doubled After controlling for vitality, faculty members with high burnout (5 or 6 on 6-pt Likert) were 2.6 times as likely (*p* = 0.01) to seriously consider leaving academic medicine than those with low burnout scores
Riddiford et al., 2017 [35] UK Cross-Sectional Survey	10-item original survey	Survey study exploring working habits and conditions of UK-based biomedical researchers (*n* = 520, 142 postdocs, 30 PIs) Findings limited to 30 Biomedical scientists. Workload: 17% reported working >70 h in the preceding week; 25% worked a 7-day week Primary faculty stressors/contributors to intent to leave: funding pressures, competing demands (teaching/research), hypercompetition, unsustainable work–life balance, and low compensation
Schindler et al., 2006 [36] USA Cross-Sectional Survey	136-item combined survey: Revised 16-item Physician Job Satisfaction Scale (PJSS) 10-item Rand Anxiety Scale (RAS) 9-item Life Satisfaction Scale (LSS) 18-item Work-Related Strain Inventory (WRSI) 20-item Center for Epidemiological Studies Depression Scale (CES-D) Open-ended comments on colleague attrition and current work/life situations	Survey study exploring the impact of work-related stressors on physical and mental health and life and job satisfaction of academic medical faculty members at 4 US medical schools (*n* = 1915, 394 Biomedical scientists) No significant differences in validated mental/physical health total scores between Clinical and Biomedical scientists: Biomedical scientists were more consistent in eating 3 meals a day, getting adequate sleep, and drinking less alcohol, but spoke to family/friends about the stressful nature of their work less often than Clinical faculty members Concerns about institutional financial health correlated most with job satisfaction in both faculty groups (*r* = −0.29–−0.41, *p* < 0.001) 1 in 5 faculty members had significant depressive symptoms, younger faculty members (27–35) reported higher total CES-D scores than older faculty members (56–65) (11.23 vs. 8.89) (*p* = 0.03) BS faculty scored significantly higher than the Clinical faculty on ‘being mentored’, ‘ability to derive personal gratification from your work’, ‘the manpower resources available to you’, ‘time allocated to teaching’, and ‘work volume’ (*p* < 0.005)
Seo et al., 2022 [37] Seo and Bae, 2024 [38] Korea Cross-Sectional Survey	21-item Maslach Burnout Inventory Human Service Survey (MBI-HS): Modified revalidated Korean version, emotional exhaustion (EE), depersonalization (DP), and personal accomplishment (PA) subscales 11-item job stress assessment (5-point Likert): managerial burden (MB), organizational pressure (OP), and daily task (DT) subdimensions	Dual publications utilizing data from a 2020 study exploring burnout and related factors in medical school faculty members from 40 medical colleges in Korea (*n* = 855, 619 Clinical, 79 Biomedical scientists) 2022 publication explored burnout prevalence and most significant stressors/sources of burnout: MBI-HS frequency of burnout (no significant differences in burnout or subscales between Biomedical scientists and Clinical faculty members; percentages represent all faculty): EE: 24.5% Moderate EE | 34.2% High EE | Mean (SD): 21.8 (10.9) DP: 13.2% Moderate DP | 66.3% High DP | Mean (SD): 15.4 (10.7) PA: 5.6% Moderate Lack of PA | 92.4% High Lack of PA | Mean (SD): 20.9 (8.5) Identified top 3 sources of burnout in both faculty groups (% agreed/strongly agreed): Excessive regulation by the government or university: (70.6% Biomedical scientists, 69.2% Clinical) Spending too many hours at work: (66.2% Biomedical scientists, 68.3% clinical) Insufficient compensation: (64.2% Biomedical scientists, 62% clinical) 37.2% of all participants identified research as the most exasperating task (no statistical difference between Biomedical scientists and Clinical). Further breakdown of stressful research tasks: - Obtaining a research grant (38% Biomedical scientists, 43.6% Clinical) - Conflicts between researchers (40.5% Biomedical scientists, 40.7% Clinical) 2024 publication explored causal relationships among job stress, burnout, and mental health Mean job stress scores (no significant differences between Biomedical scientists and Clinical faculty): Managerial burden (MB) (*t* = 0.218): Biomedical scientists: 3.77 | Clinical: 3.75 Organizational pressure (OP) (*t* = −1.009): Biomedical scientists: 3.46 | Clinical: 3.54 Daily task (DP) (*t* = −0.501): Biomedical scientists: 3.49 | Clinical: 3.54 No significant differences in mental health between Biomedical scientists and Clinical faculty Correlations: MB associated with EE (*r* = 0.621, *p* < 0.01), DP (*r* = 0.469, *p* < 0.01), PA (*r* = −0.072, *p* < 0.05), depression (*r* = 0.382, *p* < 0.01), retirement (*r* = 0.383, *p* < 0.01), and suicide (*r* = 0.128, *p* < 0.01) OP associated with EE (*r* = 0.452, *p* < 0.01), DP (*r* = 0.496, *p* < 0.01), PA (*r* = −0.193, *p* < 0.01), depression (*r* = 0.390, *p* < 0.01), retirement (*r* = 0.374, *p* < 0.01), and suicide (*r* = 0.188, *p* < 0.01) DT associated with EE (*r* = 0.538, *p* < 0.01), DP (*r* = 0.489, *p* < 0.01), PA (*r* = −0.221, *p* < 0.05), depression (*r* = 0.462, *p* < 0.01), retirement (*r* = 0.336, *p* < 0.01), and suicide (*r* = 0.117, *p* < 0.01) SEM causality assessment: job stress directly affected mental health (*β* = 0.215). Job stress affected burnout (*β* = 0.809), which in turn affected mental health (*β* = 0.609); indirect effect of job stress on mental health via burnout (*β* = 0.493)

Note: Only relevant sample sizes and findings reported.

**Table 2:** Summary of non-empirical scientific discourse.

Details	Article Type	Description
Alberts et al., 2014 [39] USA	Perspective	Discussion of systemic flaws in biomedical science and research that are threatening current and prospective careers. Contributors to distress include reductions in available funding with simultaneous institutional reliance on soft funding for salaries, hypercompetition, diminishing career opportunities with extended occupancy of trainee positions, and time demands.
Holleman et al., 2015 [8] USA/UK	Commentary	Commentary discussing sources of stress for academic biomedical scientists following interviews with department chairs at a US academic medical institution. Additionally suggests strategies to address identified concerns and stressors. Identified three major sources of stress: (1) fear of not maintaining sufficient funding, (2) frustration over administrative duties/paperwork, (3) distrust due to adversarial relationship with executive leadership.
Holleman and Gritz, 2013 [18] USA/UK	Nature Column	Career column discussing discrepancy between burnout and stress research in academic physicians vs. biomedical scientists. Discusses findings from interviews with department chairs at MD Anderson Cancer Center and a faculty survey and outlines approaches being actively implemented to address identified concerns. Primary sources of stress identified include diminishing morale due to uncertainty of career opportunities, funding, increasing standards for excellence, pressures for innovation, and administrative duties.
Watson C., 2023 [40] UK	Nature News Feature	Exploration of drivers behind the exodus of academic health researchers to industry. Identified drivers include dismal pay, hypercompetition, perceptions of replaceability, job insecurity, high workload/weekends working, administrative burden, gender inequities (particularly with funding), less funding for basic sciences (push for more translational research), and abusive behavior of senior researchers towards trainees. Reports of improved teamwork in industry with colleagues working together towards shared goals as well as better working conditions, resources, and flexibility. However, note threat to independent research due to industry influence over study design/drug use.

**Table 4:** Solution recommendations.

Challenge	Potential Solutions/Recommendation(s)
Hypercompetitive funding environment (internal and external to institution)	Increase bridge funding for biomedical scientists struggling to fund their own research [8]. Bolster institutional mechanisms to provide bridge funding, seed funding, and departmental-chair funds [8,18]. Explore approaches to decrease the % of salary that biomedical scientists are responsible for via grants [8]; improve stability through hard money [30]. Improve administrative support: grant submission process [8]; greater financial support for grant management. Improve institution/administration understanding of federal grant mechanisms and policies [30]. Increase assistance for biomedical scientists in finding nontraditional sources of research funding: philanthropic donations; indirect federal funding mechanisms [8].
Leadership conflict	Collaboration between faculty and administrative leaders; troubleshoot together [8]. Need trust-promoting leadership: more communication channels with executive level including organized meetings with faculty members to improve transparency and shared decision-making [18]. Improve mentorship and offer career counseling programs [8]. Must have sustained support from both executive and faculty leaders; supportive data can be instrumental in this effort [18].
Lack of faculty recognition	Define, structure, and align promotion and rewards systems with the roles, values, and needs of faculty members on different tracks [29]. Seek input from biomedical scientists on how to improve their work environment and work in partnership to implement the suggestions that are feasible [8,18,30].
Lack of academic career opportunities; career stagnation	Seek equilibrium in the system: align entrants to PhD training with job opportunities [academic and industry] [24]. Appropriately compensate postdoctoral fellows: increase compensation for federally funded postdocs, limit number of years that a postdoc may be supported by federal grants, and shift them into staff scientist positions with appropriate staff salaries [24]. Increase the ratio of permanent staff positions (MSc, PhD) to trainee positions [39]. Implement service structure for biomedical sciences to combat stagnation and ensure career progress [25]. Develop a Faculty Training and Mentoring Program for continuous professional development and support [25] and/or create training programs for mentors to ensure the application of evidence-based practices and promote a culture of mentoring in medical schools [5]. Increase level of formal acknowledgment of research collaboration (coauthors, coinvestigators) and include this in annual faculty appraisals and criteria for promotion [8]. Offer assistance in transitioning to alternative careers [8].
Organizational support and resources	Provide better access to mental health professionals [8] Provide training and coaching to mitigate burnout, enhance personal resilience, and foster work–life integration [8] Enhance childcare support designed to meet the professional work demands of biomedical scientists [25]

Note: Some recommendations may address multiple stressors/problems. The authors used their best judgement to filter based on the challenges each recommendation may primarily address. Counterarguments emphasizing barriers to implementation of offered solutions are not included in the current review.

## Data Availability

No new data were created or analyzed in this study. Data sharing is not applicable to this article.
